# An Analysis of Global Orthopedic Organizations Through the Lens of the Four Pillars of Global Surgery

**DOI:** 10.7759/cureus.35031

**Published:** 2023-02-15

**Authors:** Neeraj Vij, David Beyda

**Affiliations:** 1 Department of Orthopedic Surgery, University of Arizona College of Medicine-Phoenix, Phoenix, USA; 2 Department of Bioethics and Humanism, University of Arizona College of Medicine-Phoenix, Phoenix, USA

**Keywords:** equitable surgical care, low middle income country, global orthopedics, surgical equity, underserved care

## Abstract

Introduction

The mortality of orthopedic trauma is very high, however, a large proportion is considered preventable. Global orthopedics was historically centered around the direct delivery of nonsurgical and surgical medical care. There has been an evolution towards increased sustainability.

Purpose

The purpose of this paper is to evaluate the accomplishment of the four pillars of global surgery by five commonly referenced orthopedic global surgery organizations.

Methods

This institutional review board (IRB)-exempt cross-sectional data studied Global Orthopedic Alliance, Operation Rainbow, the Institute for Global Orthopaedics and Traumatology (IGOT), One World Surgery (OWS), and the Canadian Orthopedic Association for Global Surgery (COAGS) through the lens of the four pillars of global surgery: knowledge exchange, advocacy, research initiative, surgical education. The knowledge exchange pillar was examined through the three most popular online knowledge exchange platforms in orthopedics. The advocacy pillar was examined through each organization's individually created website. The research initiative was examined through a comprehensive literature search. The surgical education pillar was examined through resident-level educational resources. The data was summarized descriptively.

Results

A total of four organizations demonstrated activity within the pillar of knowledge exchange. A total of three organizations demonstrated activity with the pillar of advocacy. A total of three groups demonstrated activity within the pillar of the research initiative. A total of two groups had activity within the pillar of surgical education.

Conclusions

The landscape regarding global orthopedic surgery programs has evolved greatly to encompass the four pillars of global surgery. Within the past 10 years, there has been increased emphasis on the knowledge exchange and research initiative pillars. Surgical education remains the pillar with the least emphasis. As global orthopedic surgery programs continue to evolve, increasing emphasis should be placed on all four of these pillars to increase sustainability.

## Introduction

The leading cause of death worldwide is orthopedic trauma. Almost six million people worldwide die from traumatic injuries each year. It is estimated that up to 50% of these deaths may be preventable [1]. These numbers suggest that the annual global burden of orthopedic trauma may be larger than the combined annual burden for malaria (619,000) [2], HIV (650,000) [3], and cardiac disease (697,000) [4].

Global surgery programs first emerged in the 1980s [5]. These programs were largely focused on the delivery of both nonsurgical and surgical services to Low Middle Income Countries (LMICs) [6]. These programs were instrumental in developing the foundation of global orthopedics. They addressed the many international health issues [7] and furthered awareness regarding the need for improved international efforts towards equitable orthopedic care. These programs also decreased the burden of such trauma including those from disability, functional impairment, loss of productive human resources, and impact on quality of life [6,7]. 

In 2008, the World Journal of Surgery published an article by Dr. Farmer and Dr. Kim in which they described global surgery as the “neglected stepchild of global health” [8]. In this article, these authors called on a commitment towards serving the world’s poorest, the integration of “vertical” surgical missions, and the building of infrastructure. In recent years, orthopedic global surgery organizations have moved towards practicing in cost-efficient and sustainable mechanisms.

Modern global surgery has evolved outside the direct delivery of surgical care to include four pillars: knowledge exchange, advocacy, research initiative, and surgical education [9]. These four pillars are centered around a heightened sense of the importance of sustainability. The purpose of this paper is to utilize the four pillars to evaluate five commonly referenced orthopedic global surgery organizations: Global Orthopedic Alliance, Operation Rainbow, the Institute for Global Orthopedics and Traumatology (IGOT), One World Surgery, and Canadian Orthopedic Association Global Surgery (COAGS).

## Materials and methods

General

This study was determined exempt per the determination of 'Not Human Subjects Research as defined by 45 CFR 46.102(e)' by the University of Arizona Institutional Review Board. This was a cross-sectional study and data collection was performed on the date January 5th, 2023.

We studied the reach of five common orthopedic global surgery organizations as defined by four pillars 1) knowledge exchange, 2) advocacy, 3) research initiative, 4) surgical education. The knowledge exchange pillar was examined through the three most popular online knowledge exchange platforms in orthopedics (Table 1).

**Table 1:** The three commonly used surgical exchange platforms searched to study pillar 1, knowledge exchange.

Surgical exchange platform	Surgical exchange website	
VuMedi	https://www.vumedi.com/orthopaedics/	
Medtube	https://medtube.net/orthopedics	
		Video Journal of Orthopaedics	https://www.vjortho.com/	

The advocacy pillar was examined through each organization's individually created website (Table 2).

**Table 2:** Five common orthopedic global surgery organizations examined in our study.

Organization	Official website
Global Orthopedic Alliance	https://www.hss.edu/global-partnerships.asp
Operation Rainbow	https://operationrainbow.org/
Institute for Global Orthopaedics and Traumatology (IGOT)	https://orthosurgery.ucsf.edu/outreach/global/igot_program
One World Surgery	https://oneworldsurgery.org/
Canadian Orthopaedic Association Global Surgery (COAGS)	https://coa-aco.org/coa-global-surgery-coags/

The research initiative was examined through a comprehensive literature search (details below). The surgical education pillar data collection process was two-fold. Firstly, the organizations individually created websites were searched for relevant subcomittees in this pillar. Secondly, any references identified from the online exchange platforms that were specifically resident level educational resources were also included in this pillar.

Literature search (research initiative pillar)

The following search engines were used: Medline, PubMed Advanced Search, Cochrane library, Embase, and Scopus. Cochrane Reviews was also searched per the recommendations of Pautasso M [10]. The following search items were used: ‘global surgery’ OR ‘global orthopedics’ OR ‘LMIC’ OR ‘Low Middle Income Country’ OR ‘orthopedic trauma’ OR ‘traumatology’ AND ‘[Organization Name]’ OR ‘[Organization Abbreviation]’. The Organization Name and Organization Abbreviation fields were trialed through the following: Global Orthopedic Alliance, Operation Rainbow, Institute for Global Orthopedics and Traumatology + IGOT, One World Surgery, Canadian Orthopedic Association Global Surgery + COAGS.

Articles published between January 1975 to December 2021 were included. All of the articles were screened by title and abstract. A preliminary decision to include or exclude an article was made based on the relevance of the information within the abstract with regard to the organization being studied and the topic of global orthopedics. This resulted in a preliminary list of 634 articles. Of these, a total of 57 articles were determined as contributory to the research initiative pillar.

Funding statement

This research did not receive any specific grant from funding agencies in the public, commercial, or not-for-profit sectors.

Data collection and abstraction

With regards to pillar 1) knowledge exchange, the following data were collected: name of any relevant subcommittees within the organization of study, the earliest date of occurrence number of occurrences regarding knowledge exchange, and the number of views per occurrence. The average and mean of the number of views were calculated. With regards to pillar 2) advocacy, the following data were collected: name of any relevant committees, qualitative description of the purpose, and earliest date of occurrence. With regards to pillar 3) research initiative, the following data were collected: name of any relevant committees, number of publications regarding global orthopedics containing organization as an affiliation, and earliest date of occurrence. With regards to pillar 4) surgical education, the following data were collected: name of any relevant committees, number of views per occurrence, and earliest date of occurrence. The results were summarized descriptively and tabulated using Microsoft Excel Version 1.66.1.

## Results

A total of four organizations demonstrated activity within pillar 1) knowledge exchange (Table 3).

**Table 3:** The results pertaining to pillar 1) Knowledge Exchange.

Organization	Name of the relevant committee	Earliest year of occurrence	Number of occurrences	The average number of views per occurrence	
					Global Orthopedic Alliance	Orthopedic Knowledge Network	N/A	/NA	N/A	
Operation Rainbow	N/A	N/A	N/A	N/A	
Institute for Global Orthopaedics and Traumatology (IGOT)	IGOT Portal	2006	98	534	
One World Surgery	N/A	2012	65	356	
Canadian Orthopaedic Association Global Surgery (COAGS)	N/A	2014	27	231	

Two of these groups had distinctly named committees or subgroups dedicated to this pillar. The earliest date of occurrence was 2006. The average number of views ranged from 231 to 534. The number of occurrences ranged from 27 - 98. A total of three organizations demonstrated activity with pillar 2) advocacy (Table 4). Two of these groups had distinctly named committees or subgroups dedicated to this pillar. The earliest date of occurrence was 2006.

**Table 4:** The results pertaining to pillar 2) Advocacy.

Organization	Name of the relevant committee	Earliest date of occurrence	Qualitative description of purpose
Global Orthopedic Alliance	Humanitarian Initiatives Program	2014	The Humanitarian Initiatives Program addresses the advocacy pillar by supporting projects aimed at increasing local and international resources available to Low Middle Income Countries and by promoting the needs of local communities.
Operation Rainbow	N/A	N/A	N/A
Institute for Global Orthopaedics and Traumatology (IGOT)	COACT	2006	The Consortium of Orthopaedic Academic Traumatologists (COACT) supports the advocacy pillar by promoting the most recent advances in global surgery practices and development of scholarly efforts aimed particularly at voicing community needs. The COACT uses a large platform including many well recoginzed international institutions that allow for a communicative and collaborative spirit and thus facilitate the advocacy of the needs in local communities in Low Middle Income Countries.
One World Surgery	N/A	N/A	N/A
Canadian Orthopaedic Association Global Surgery (COAGS)	N/A	N/A	N/A

A total of three groups demonstrated activity within pillar of 3) research initiative (Table 5). Only one of these groups had a distinctly named committee or subgroup as dedicated to this pillar. The earliest date of occurrence was 2007.

**Table 5:** The results pertaining to pillar 3) Research Initiative.

Organization	Name of the relevant committee	Earliest date of occurrence	Number of institutional publications
Global Orthopedic Alliance	N/A	2014	17
Operation Rainbow	N/A	N/A	N/A
Institute for Global Orthopaedics and Traumatology (IGOT)	Global Research Initiative	2007	32
One World Surgery	N/A	N/A	N/A
Canadian Orthopaedic Association Global Surgery (COAGS)	N/A	2012	8

The number of institutional publications ranged from eight to 17. A total of two groups demonstrated activity within pillar 4) surgical education (Table 6). Two of these groups had a distinctly named committee or subgroup as dedicated to this pillar. The earliest date of occurrence was 2006.

**Table 6:** The results pertaining to pillar 4) Surgical Education.

Organization	Name of the relevant committee	Earliest year of occurrence
Global Orthopedic Alliance	eAcademy	2014
Operation Rainbow	N/A	N/A
Institute for Global Orthopaedics and Traumatology (IGOT)	Surgical Management and Reconstructive Training (SMART) course	2006
One World Surgery	N/A	N/A
Canadian Orthopaedic Association Global Surgery (COAGS)	N/A	N/A

## Discussion

This was a cross-sectional study that examined the current state of five common orthopedic global surgery organizations through the four pillars of global surgery. We found that the pillars of knowledge exchange and research initiative were well represented among all five of the organizations studied. The pillar of surgical education continues to remain the least well represented in global orthopedics today.

Global orthopedics has evolved greatly since its advent. Some of the earliest organizations in global orthopedics include orthopedic overseas [6] and world orthopedic concern [7]. These programs were instrumental in establishing the subdiscipline of global orthopedics [11] and emphasized the delivery of nonsurgical and surgical care [6,7]. World orthopedic concern, in particular, was instrumental in the battle against polio and is still revered today by many nations for the long-lasting effect it has had [11]. Orthopedic Overseas was another such organization [6]. This organization provided much of the framework for the delivery of orthopedic care to underserved nations that is performed today [7]. In particular, this organization voiced the need for this work towards the orthopedic community at large [12]. However, since the advent of these instrumental organizations, global orthopedics has evolved greatly.

The results of our study demonstrate a heightened understanding of the importance of sustainability in global health. We noted a representation of at least three pillars of global orthopedics in three of the five organizations studied. The earliest occurrences can be seen in Tables 3-6. One can note that the increase in representation of these pillars is largely seen within the last 10 to 15 years.

Our study revealed a good representation of the knowledge exchange pillar. A total of three organizations demonstrated commitment to this pillar. In 2005, Yang et al. [13] argued for a redirection with regard to sustainability in global health. They proposed a new definition of sustainability, in particular, one that does not require consumable medical interventions to provide health [13]. A key component of this is the distribution of knowledge to LMICs in a way that promotes sustainable growth. The results of our study demonstrate improvement in global orthopedics through this lens. Global orthopedics has made some progress considering the increase in representation of the knowledge exchange and research initiative pillars. These represent facets of global surgery that would allow for increased education of the providers in LMICs and thus inherently increase the surgical capacities in these nations.

A good representation of the research initiative pillar was also noted in our study with three pillars demonstrating activity within this pillar. There has been a push towards increased academic efforts that better address the needs of LMICs [12,14,15]. Wendler et al. [14] highlight several very important points, in particular, that such research should both have relevance and sufficient impact on the communities that we intend to serve. The results of our study demonstrated an improvement in this domain considering our methodology that required relevance to the nation of interest. However, further scholarly efforts from global orthopedic organizations should be further focused on the points brought up by Wendler et al. [14].

A key component of equitable surgical care is the production of equally trained surgical residents in LMICs [16]. Advancing surgical training worldwide has the potential to improve the delivery of equitable and safe surgery with a cumulative effect that is far superior to any single medical mission [16]. However, prior studies have shown that postgraduate surgical training varies greatly in LMICs [17] and can lack appropriate specificity with regard to surgical specialties [18]. The results of our study are in concordance with this finding with two of the five organizations studied demonstrating some representation of the surgical education pillar. The evaluation of this pillar was two-fold and involved both, searching the organization's website for activity in this pillar and searching for specific resident level resources on the online exchange platforms. One postulation as to the relatively weaker representation of this pillar is with regard to our foundation in global orthopedic surgery. A large majority of the initial efforts in global orthopedics were centered around ongoing issues that required acute intervention [17,18].

Of note to this conversation is the Surgical Management and Reconstructive Training developed by IGOT. This organization demonstrates the excellent representation of the production of equally trained surgeons in LMICs. However, as the field of global orthopedics continues to grow alongside the principle of sustainability, similarly focused organizations should aim to increase the representation of this pillar. A valuable tool in doing so is free publicly available online courses that are supplemented with in-house training. This not only fosters the growth of the surgical education pillar, but also the development of long-term relationships that will serve the field well.

This study is not without its limitations. As a cross-sectional study it is unable to draw inferences regarding the progress of orthopedic global surgery without the use of priorly published literature. Further, the data collection process was limited to that conducted by two authors at a single institution, though both of these authors have sufficient experience in global health research. Lastly, this article is limited to the viewpoint of the priorly published four pillars of global surgery; however, other lenses may exist by which one can understand the reach of these organizations.

## Conclusions

The landscape regarding global orthopedic surgery programs has evolved greatly to encompass the four pillars of global surgery. Within the past 10 years, there has been increased emphasis on the knowledge exchange and research initiative pillars. Surgical education remains the pillar with the least emphasis. Orthopedic global surgery organizations should move towards increased utilization of free online platforms with supplemental hands-on training to improve surgical education in LMICs. As global orthopedic surgery programs continue to evolve, increasing emphasis should be placed on all four pillars to increase sustainability.
